# Simulation of Bullet Fragmentation and Penetration in Granular Media

**DOI:** 10.3390/ma13225243

**Published:** 2020-11-20

**Authors:** Froylan Alonso Soriano-Moranchel, Juan Manuel Sandoval-Pineda, Guadalupe Juliana Gutiérrez-Paredes, Usiel Sandino Silva-Rivera, Luis Armando Flores-Herrera

**Affiliations:** 1 Postgraduate Studies and Research Section, Instituto Politecnico Nacional, Higher School of Mechanical and Electrical Engineering, U. Azcapotzalco, Av. Granjas 682, Mexico City 02250, Mexico; fsorianom0800@alumno.ipn.mx (F.A.S.-M.); jsandovalp@ipn.mx (J.M.S.-P.); ggutierrezp@ipn.mx (G.J.G.-P.); 2 SEDENA, D.G.E.M., Rectory of the Army and Air Force University, Escuela Militar de Ingenieros, Av. Industria Militar 261, Naucalpan de Juarez 53960, Estado de Mexico, Mexico; ussilvar@ing-mil.com

**Keywords:** impact, sand, bullet penetration, granular media, energy dissipation, transient analysis

## Abstract

The aim of this work is to simulate the fragmentation of bullets impacted through granular media, in this case, sand. In order to validate the simulation, a group of experiments were conducted with the sand contained in two different box prototypes. The walls of the first box were constructed with fiberglass and the second with plywood. The prototypes were subjected to the impact force of bullets fired 15 m away from the box. After the shots, X-ray photographs were taken to observe the penetration depth. Transient numerical analyses were conducted to simulate these physical phenomena by using the smooth particle hydrodynamics (SPH) module of ANSYS^®^ 2019 AUTODYN software. Advantageously, this module considers the granular media as a group of uniform particles capable of transferring kinetic energy during the elastic collision component of an impact. The experimental results demonstrated a reduction in the maximum bullet kinetic energy of 2750 J to 100 J in 0.8 ms. The numerical results compared with the X-ray photographs showed similar results demonstrating the capability of sand to dissipate kinetic energy and the fragmentation of the bullet caused at the moment of impact.

## 1. Introduction

The analysis of bullet penetration through different materials is an important issue to achieve adequate safety conditions during the design of containers, pressure vessels, vehicles, armor and other similar products, especially barricades, to protect security forces. These studies have been performed in previous works considering steels and aluminum alloys [1,2,3]. Børvik carried out several tests with 6082-T651 and 6082-T4 aluminum plates with changes in the angle of impact and plate characteristics [4,5]. In specific defense situations, the behavior of the material supporting bullet impacts is analyzed depending on the crater formation, for example, in continuous media [6]. However, granular media is another option to reduce the kinetic energy of a bullet over short distances [7,8], especially when such media are used in the form of ballistic blocks. The granular nature of sand, its recycling capability combined with its complex interaction dynamics creates unique physical capabilities. The mixture of sand with other construction materials for example, creates important characteristics suitable for several mechanical and construction applications [9,10,11,12,13,14,15,16,17,18,19,20,21,22,23]. This phenomenon has been studied here as an alternative to achieve the goal of bullet fragmentation. Several containers constructed with fiberglass and considering different geometries have been used as an alternative to constructing ballistic walls [24,25,26,27,28,29,30]. Based on these works, samples of ballistic walls were constructed in this research to observe the behavior of a bullet when entering a coupled system. This system is formed by continuous media as the initial impact material and sand as the granular media. The purpose of the present study is to measure the penetration distance of a bullet impacting the materials considered in the continuous system, which are plywood and fiberglass shells. The numerical and experimental results obtained with the combination of materials used for the construction of continuous and granular media are the main contributions of this study. A numerical analysis was carried out using ANSYS^®^ 2019R1-AUTODYN to simulate the behavior of the bullet when impacting the samples and to obtain the magnitudes of the kinetic energy absorbed by the coupled system [31]. The dynamic behavior of the granular media as a group of uniform particles transferring the kinetic energy when receiving the 7.62 mm projectile was simulated using the smooth particle hydrodynamics (SPH) model, together with the compaction equations of state (EOS) and the model option MO granular. This transient analysis includes the interaction of the particles inside the coupled system when receiving the impact; in some cases, the bullet deviates with respect to the original trajectory, and in other cases, the bullet is destroyed because of the instantaneous heat and mechanical friction [32].

## 2. Materials and Methods

### 2.1. Ballistic Blocks

Two types of block prototype were built for the experiment, one that forms the plywood-sand-plywood (PSP) system and another that forms the fiberglass-sand-fiberglass (FSF) system. The construction criteria for the block walls considered the minimum commercial thickness walls required to support 10 kg of sand per block, but also allowing stacking up to 7 blocks to form a 2.1 m high ballistic wall without presenting structural instabilities. As a result of the tests, the resulting wall for the PSP system was 12 mm and for the FSF system it was 6 mm. Finally, a total of 6 blocks were built for each system. The final dimensions of the blocks were 300 × 300 × 300 mm in length, width and height, respectively. The experiment considered beach-type sand which was first passed through a sieve to eliminate the presence of stones, garbage and other solid objects and it was left to dry in the sun for 10 days, moved with a shovel and covered at night for the purpose of removing traces of moisture. It is very important to remove moisture before the experiments to maintain uniform distribution of the granular media and to avoid a lubricating effect during the friction between the sand and the bullet.

### 2.2. Ballistic Workbench

**Figure 1:**
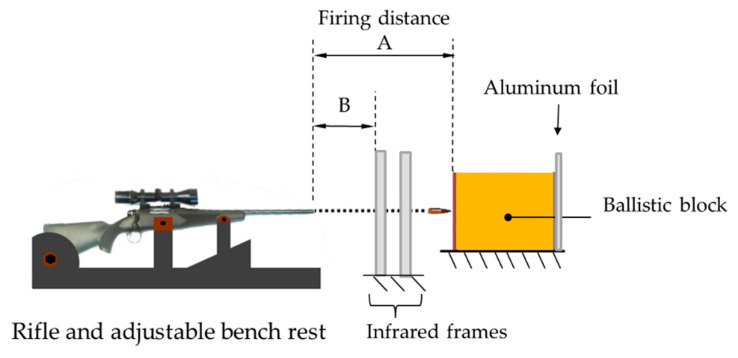
Configuration of the ballistic workbench.

Figure 1 shows the components of the experimental setup. Two types of bullets were used for the experiments, the Full Metal Jacket (FMJ) and the Armor Piercing (AP). An Oehler ballistic chronograph with optical barriers was used to obtain pressure, energy and velocity values. In the figure, the Marlin XL7 rifle is shown on the left side and the ballistic block is located 15 m away on the right side (distance A). Between the rifle and the ballistic block, a pair of infrared frames were located to measure the velocity of the bullet. The first infrared fame which was the start trigger was located 12 m away from the rifle (distance B). The second infrared frame was the stop trigger and it was located 1.5 m from the first one. The rifle was mounted on an adjustable rifle bench rest located 15 m away from the target. This distance reduced the possibility of nutation, yaw or precession of the bullet before the impact. The rifle bench rest was adjusted and used in a fixed position for every shot. Behind the ballistic block, a layer of 0.2 mm of aluminum foil was located to verify the presence of any remaining fragment after the shots. X-ray photographs were taken on the ballistic blocks after the shots with a Vertex II equipment (VJ Technologies, Inc., Suffolk County, NY, USA), with capacity of 160 kV.

**Figure 2:**
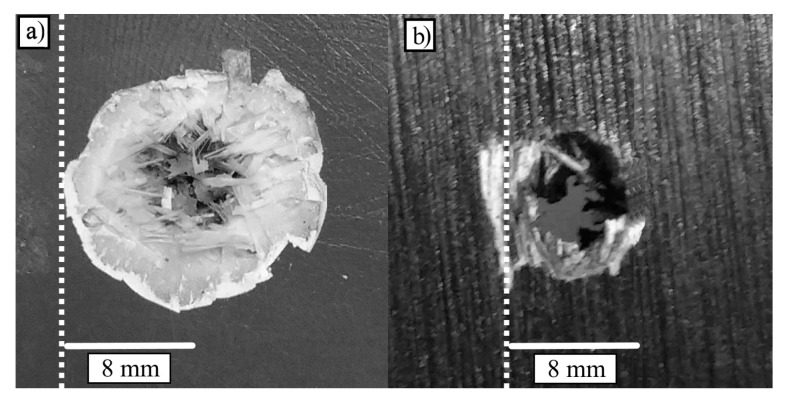
Resulting crater of the 7.62 mm FMJ (Full Metal Jacket) bullet impacted in (**a**) the fiberglass-sand-fiberglass (FSF) system and (**b**) the plywood-sand-plywood (PSP) system.

Figure 2 shows a close view of the perforations produced by the impacts in each system. The circular shape of the craters show that the bullet has penetrated without inclination, this means the longitudinal axle of the bullet enters perpendicular with respect to the wall surface. It can be seen in (a) the brittle fracture of the FSF system and in (b) the ductile hole growth of the PSP system.

Table 1 shows the obtained velocities measured as indicated in “Cartridge, 7.62 mm: NATO, Ball-M80MIL-DTL-46931” standards for FMJ M80 bullets and “MIL-C-60617A” for armor-piercing bullets.

### 2.3. Numerical Simulation

**Figure 3:**
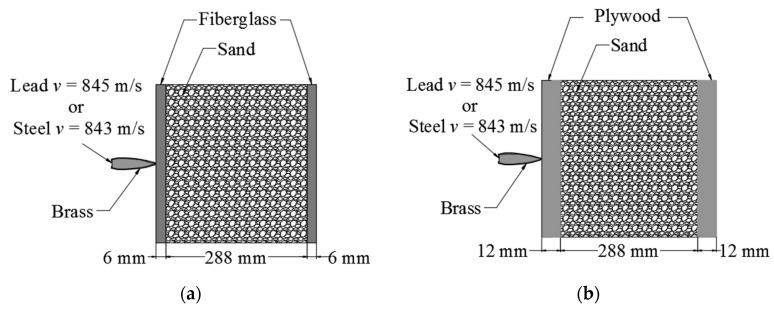
Components of the ballistic blocks in (**a**) the FSF system and (**b**) the PSP system.

3D-CAD models were constructed for the numerical analysis simulation to observe the penetration depth of the bullet through the constructed systems and compare the resulting values with the experimental test. Figure 3 shows the corresponding diagram for each system. Two sidewalls represent the continuous media, and a region between the walls represents the granular media. Figure 3a shows the FSF system, and Figure 3b shows the PSP system. The bullet is located with the longitudinal axis perpendicular to the surface of the wall, and effects of nutation, yaw or precession are not included.

The AP (M61) and FMJ (M80) bullets contain an external jacket of brass. The left side of each block shows the location of the bullet with initial velocities of 845 m/s and 843 m/s for the lead and steel cores, respectively. The material properties considered for the simulation are shown in Table 2.

With respect to the boundary conditions, the ballistic block models were subjected to the same restricted conditions; the horizontal displacement of the external faces of the continuous media was restricted in the form of fixed support along the *Z*-axis but not for the remaining perpendicular directions (X and Y). The creation of the resulting mesh for each model was also the result of improved trials to achieve suitable values for the orthogonal and skew qualities. To improve in the simulation time, the bullet was located 1 mm away from the contact face, with one small timestep simulated before the impact. The simulation scenarios were divided into 4 different cases, as shown in Table 3: two for an initial velocity of 843 m/s and two for 845 m/s.

**Figure 4:**
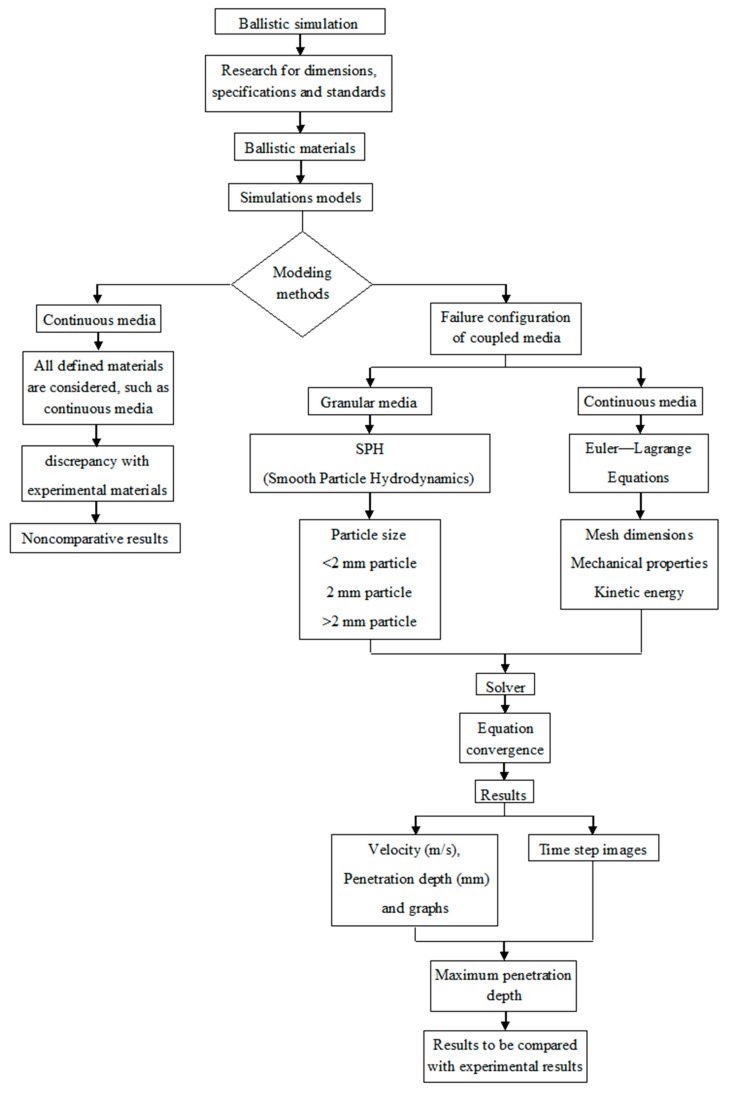
Simulation process flowchart.

Figure 4 shows the simulation process flowchart as a result of the previous research conducted for the construction of the models. The behavior of continuous media considers all the material particles interconnected and working as a single piece in comparison with granular media in which the mechanical property of the particle interacts and reorganizes during the dynamic process.

This configuration is a combination of selected parameters that involve the material models available in the software (ANSYS^®^ 2019R1, Canonsburg, PA, USA), and the constants were taken from the AUTODYN library. The Smoothed Particle Hydrodynamics Lagrangian particle method (SPH) of ANSYS^®^ AUTODYN 2019R1 was selected for this simulation, the continuous media is considered as a uniform set of particles interacting with the energies created during the evolution of the impact. Multilinear isotropic hardening behavior was also selected to establish the plastic behavior of this material [33,34,35,36,37,38].

In the simulation, the effect caused by the angular velocity of the bullet is not considered, and because of that, it was expected that there would be differences between the experimental and numerical simulation results. The mechanical behavior of the lead and steel, which are the core materials, were included by considering the Steinberg–Guinan strength formulation. The fiberglass was modeled with the Johnson–Holmquist continuous-strength formulation and the plywood was modeled considering the polynomial Equation of State (EOS) formulation. The behavior of the granular media was modeled as MO Granular Failure Model together with tensile pressure failure and the walls were configured with the shock EOS linear formulation [39,40,41,42,43]. This combination allows the granular media to transfer the load vectors and reorganize the media during the displacement of the bullet. The Grüneisen parameter is conventionally written as a dimensionless combination of the expansion coefficient, bulk modulus, density and specific heat and can also be presented in terms of elastic moduli and their pressure derivatives, providing a quantitative link between thermal and mechanical parameters [44]. The material properties and constant values considered for the simulation are shown in Table 4 [25,32,45,46].

This combination of parameters, as shown below, allowed us to observe the fragmentation of the bullet caused by the initial contact with the walls which is of special importance in terminal ballistics and military medicine [47,48,49].

## 3. Results and Discussion

**Figure 5:**
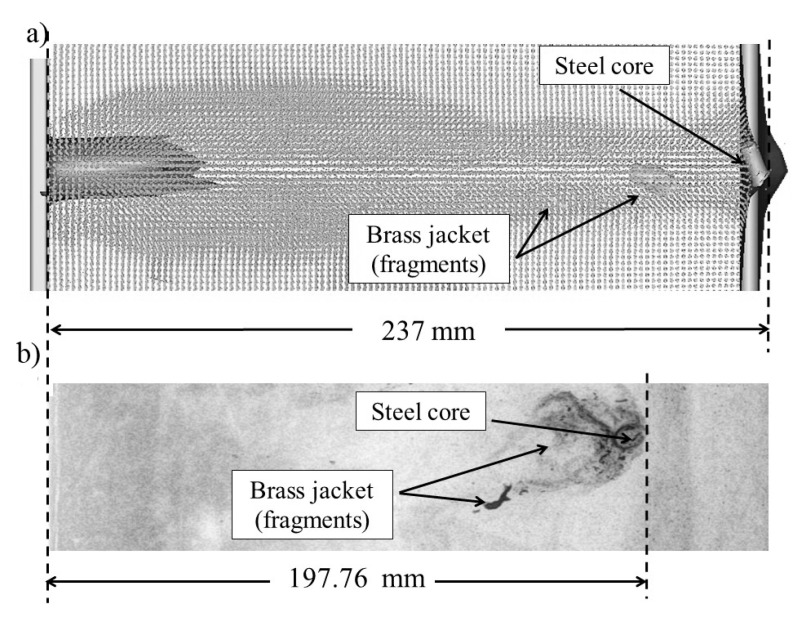
Comparison of fragmentation and penetration depths for the 7.62 mm AP bullet in the FSF block from (**a**) numerical simulation and (**b**) X-ray photography.

Figure 5 shows (a) the results of the numerical simulation compared with (b) the experimental X-ray photography. This figure corresponds to the FSF AP M61 block (case 1). The numerical simulation predicted a penetration distance of 237 mm and the appearance of compaction waves along the penetration trajectory showing fragmentation of the brass after 204 mm. In the simulation and the X-ray photograph, an expanded distribution of the compacting wave is observed [50]. Even when the final penetration distances are not the same, both circumstances show brass jacket fragmentation before the steel core stops. The steel core showed an additional advance of at least 30 mm ahead. In the X-ray photography, a maximum fragmentation of the brass was 148 mm, and an additional advance of the steel core of 49 mm which gives a total depth of 197.76 mm. In both analyses, the final position of the bullet rotated with respect to the initial entry orientation.

**Figure 6:**
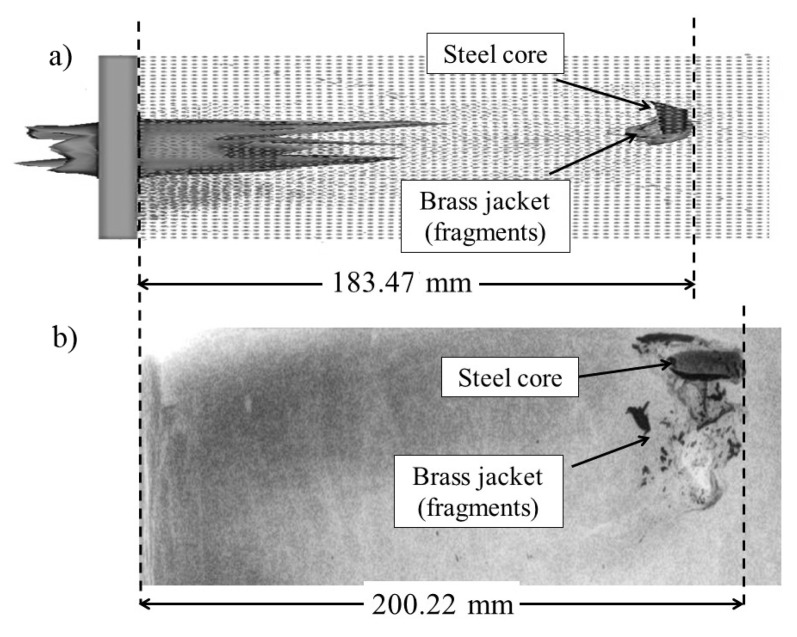
Comparison of fragmentation and penetration depths of the 7.62 mm AP bullet in the PSP block from (**a**) numerical simulation and (**b**) X-ray photography.

The results of the fragmentation and penetration distance reached by the 7.62 mm AP projectile for the PSP AP M61 block are shown in Figure 6 (case 2). In (a) the numerical simulation and (b) the X-ray photography, the maximum penetration distance is 183.47 and 200.22 mm, respectively. The X-ray photography shows more concentrated compacting wave in the upward direction following the bullet trajectory and the final location of the fragments are very close to the core.

**Figure 7:**
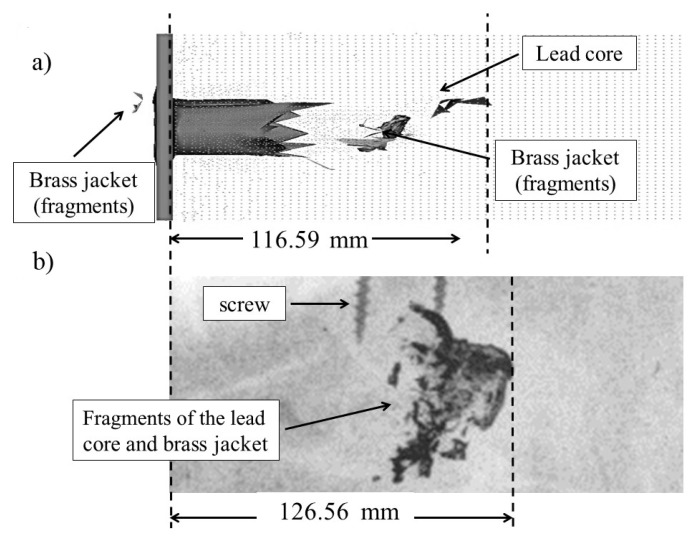
Comparison of fragmentation and penetration depths of the 7.62 mm FMJ bullet in the FSF block from (**a**) numerical simulation and (**b**) X-ray photography.

Figure 7 shows the results of (a) the numerical simulation and (b) X-ray photography for the 7.62 mm FMJ M80 projectile impacting the FSF block (case 3). In the simulation, a penetration distance of 116.59 mm was obtained, and in the X-ray photography, a measured distance of 126.56 mm was obtained. A wide compaction wave is located ahead the core and the expanded fragments showing a reduced penetration distance with respect to the previous cases.

**Figure 8:**
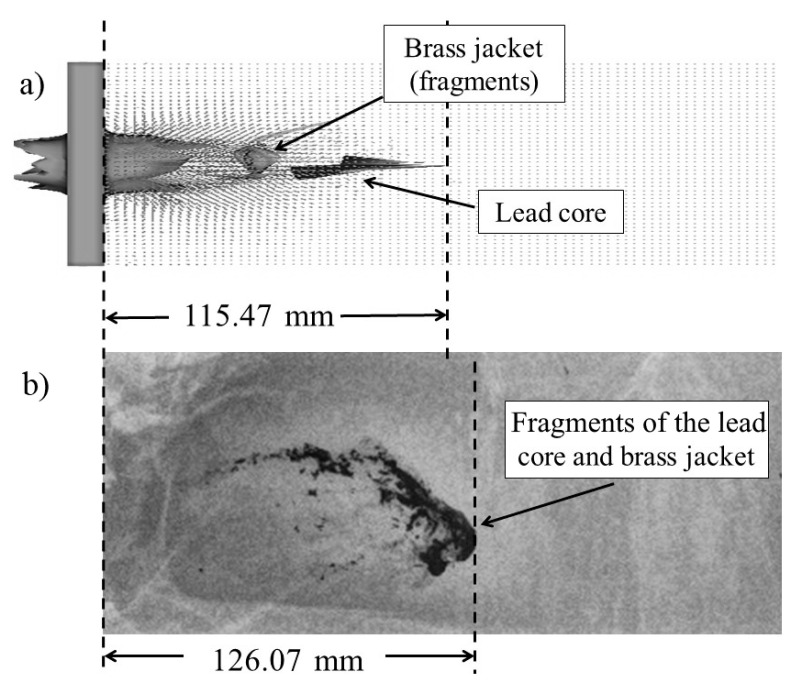
Comparison of fragmentation and penetration depths of the 7.62 mm FMJ bullet in the PSP block from (**a**) numerical simulation and (**b**) X-ray photography.

Figure 8 shows the results of case 4 in which an FMJ M80 bullet impacts the PSP box. The penetration distances obtained in (a) the numerical simulation and (b) the X-ray photography were 115.47 and 126.07 mm, respectively. A unique zone of extreme fragmentation was not identified but was the most extended instead.

**Figure 9:**
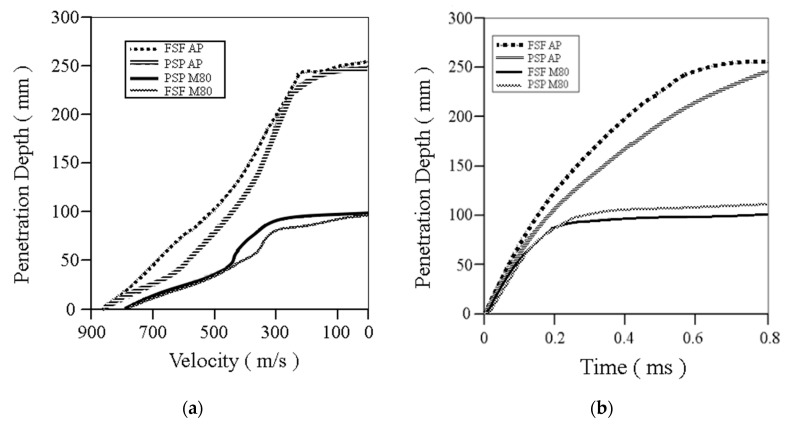
Penetration depth with respect to (**a**) velocity and (**b**) time.

Figure 9a shows the numerical results of the penetration depth with respect to the velocity starting from the penetration velocity for each of the four cases, and Figure 9b shows the penetration depth with respect to time.

**Figure 10:**
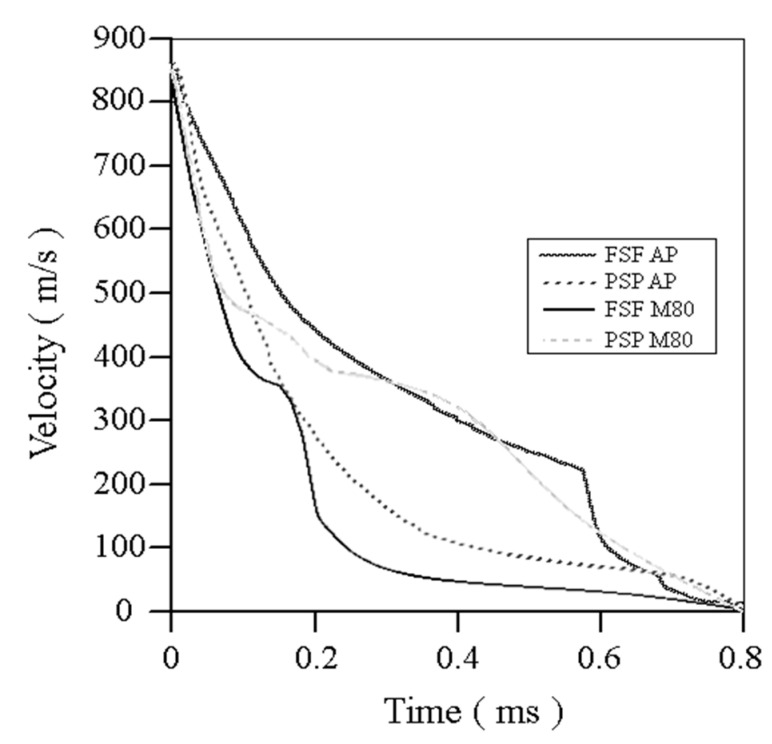
Numerical velocity results with respect to time obtained in the numerical simulations.

Figure 10 shows the dependence of the bullet’s velocity inside the blocks with respect to time. In all cases, the velocity is zero after 0.8 ms. Although the spinning effect of the bullet in the simulation is not considered, the hardness and ordering of each wall material has an important effect on penetration. In the case of fiberglass, the fibers do not have a unique arrangement and the energy absorption dissipates in random directions at each instant. As the bullet advances through the fiberglass, it encounters new fibers every layer reacting together with the neighboring fibers. In the plywood, the fibers are arranged in the same direction and they are all compressed together at the same time, deforming and deflecting the energy in a single direction. This also causes an opening of the fibers due to the spinning effect and allowing the bullet to advance with greater force than in the case of the fiberglass wall.

Table 5 shows the numerical values obtained in the numerical analyses compared with the experimental results, the shortest stopping distance was found for case 4. This minimum value was obtained in the numerical simulation as well as in the experimental measurements.

## 4. Conclusions

During the impact, the bullet entered first into a continuous medium of wood with a thickness of 12 mm and the penetration depth was reduced up to 50.48% compared to the distance obtained without the shells. A variation of 7.53 m/s between the experimental results and numerical results was found. The numerical and experimental results showed similar fragmentation distribution of the bullets with 95.34% similarity in the penetration distance of the FMJ projectile and 88.63% for the AP projectile. The impact surface areas were 100 mm^2^ and 300 mm^2^ with a depth of 300 mm. The numerical analysis was solved with the SPH module of ANSYS^®^ Autodyn. It was determined that the initial impact of this type of projectile through a continuous medium, such as the 12 mm wood plate, reduces the penetration capacity by up to 48.62%. The presented fragmentation and contention of the bullet material can represent a tactical advantage since the impact of the 7.62 mm AP projectile reaches a maximum penetration distance of 52 mm compared with 100 mm obtained without the thick initial continuous medium. The use of the fibrous materials provides the advantage that they are cheap, eco-friendly and easy to repair compared to other ballistic blocks based on ceramics and plastics which once fractured cannot be repaired and, in some cases, they must be discarded.

## Figures and Tables

**Table 1:** Bullet velocities obtained in the experimental shots.

Bullet Type	Weight (±0.01 g)	Energy at 15 m	Velocity at 0 m	Velocity at 15 m	Velocity at 23.77 m
AP M61	9.75 g	3 481 J	852 m/s	843 m/s	835 m/s
FMJ M80	9.65 g	3 445 J	855 m/s	845 m/s	839 m/s

**Table 2:** Mechanical properties required in the simulation.

Material	Media	Material	Density (kg/m^3^)	Shear Modulus (GPa)
Brass	Continuous	Orthotropic	8450	35.9
Lead	Continuous	Orthotropic	11,350	4
Steel	Continuous	Orthotropic	7896	81.8
Sand	Granular	Anisotropic	2641	76.9
Plywood	Continuous	Anisotropic	680	0.75
Fiberglass	Continuous	Anisotropic	1310	0.82

**Table 3:** Bullet velocities considered for each case of analysis.

Case	Model Type–Projectile	Velocity (m/s)
1	FSF–AP (M61)	843
2	PSP–AP (M61)	843
3	FSF–FMJ (M80)	845
4	PSP–FMJ (M80)	845

**Table 4:** Configuration parameters for the simulation.

	Lead	Brass	Steel	Sand	Fiberglass	Plywood
Shock EOS Linear	-	-	-	X	-	X
Grüneisen Coefficient	2.74	2.04	2.17	X	1.18	X
C1 (m/s)	2006	3726	4569	X	2746	X
S1	1.429	1.434	1.49	X	1.319	X
Quadratic S2 (s/m)	0	0	0	X	0	X
Specific Heat (J/kg C)	124	X	447	X	X	X
Steinberg Giunan Strength	-	X	-	X	X	X
MO Granular	X	X	X	-	X	X
offset	X	X	X	0	X	X
Tensile Pressure Failure	X	X	X	-	X	X
Max. Tensile Pressure (Pa)	X	X	X	1000	X	X
Compaction EOS Linear	X	X	X	-	X	X
Solid Density (kg/m^3^)	X	X	X	2641	X	X
Compaction Path	X	X	X	-	X	X
Linear Unloading	X	X	X	-	X	X
Johnson-Holmquist Strength	X	X	X	X	X	-
Failure type	X	X	X	X	X	Gradual
Hugoniot Elastic Limit	X	X	X	X	X	5.92 × 10^9^ Pa
Intact Strength Constant A	X	X	X	X	X	0.93
Intact Strength Exponent N	X	X	X	X	X	0.77
Strain Rate Constant C	X	X	X	X	X	0.003
Fracture Strength Constant B	X	X	X	X	X	0.088
Fracture Strength Exponent m	X	X	X	X	X	0.35
Max. fracture strength Ratio	X	X	X	X	X	0.5
Damage constant D1	X	X	X	X	X	0.053
Damage constant D2	X	X	X	X	X	0.85
Bulking constant B	X	X	X	X	X	1
Hydrodynamic Tensile Limit	X	X	X	X	X	−0.15 × 10^9^ Pa
Bulk Modulus	X	X	X	X	X	45.4 × 10^9^ Pa
Shear Modulus	X	X	X	X	X	15,000 MPa
Polynomial EOS	X	X	X	X	X	-

**Table 5:** Comparison of numerical and experimental results for each case.

Case	Model Type–Projectile	Velocity (m/s)	Penetration Depth (mm)		Time (ms)
			Numerical	Experimental	
1	FSF–AP (FMJ)	843	237.12	197.76	0.8
2	PSP–AP (FMJ)	843	207.41	200.22	0.8
3	FSF–FMJ (M80)	845	116.59	126.56	0.8
4	PSP–FMJ (M80)	845	115.35	126.07	0.8
